# Predicting River Macroinvertebrate Communities Distributional Shifts under Future Global Change Scenarios in the Spanish Mediterranean Area

**DOI:** 10.1371/journal.pone.0167904

**Published:** 2017-01-30

**Authors:** Javier Alba-Tercedor, Marta Sáinz-Bariáin, José Manuel Poquet, Roberto Rodríguez-López

**Affiliations:** Department of Zoology, University of Granada, Granada, Spain; University of Delhi, INDIA

## Abstract

Several studies on global change over the next century predict increases in mean air temperatures of between 1°C to 5°C that would affect not only water temperature but also river flow. Climate is the predominant environmental driver of thermal and flow regimes of freshwater ecosystems, determining survival, growth, metabolism, phenology and behaviour as well as biotic interactions of aquatic fauna. Thus, these changes would also have consequences for species phenology, their distribution range, and the composition and dynamics of communities. These effects are expected to be especially severe in the Mediterranean basin due its particular climate conditions, seriously threatening Southern European ecosystems. In addition, species with restricted distributions and narrow ecological requirements, such as those living in the headwaters of rivers, will be severely affected. The study area corresponds to the Spanish Mediterranean and Balearic Islands, delimited by the Köppen climate boundary. With the application of the MEDPACS (MEDiterranean Prediction And Classification System) predictive approach, the macroinvertebrate community was predicted for current conditions and compared with three posible scenarios of watertemperature increase and its associated water flow reductions. The results indicate that the aquatic macroinvertebrate communities will undergo a drastic impact, with reductions in taxa richness for each scenario in relation to simulated current conditions, accompanied by changes in the taxa distribution pattern. Accordingly, the distribution area of most of the taxa (65.96%) inhabiting the mid-high elevations would contract and rise in altitude. Thus, families containing a great number of generalist species will move upstream to colonize new zones with lower water temperatures. By contrast, more vulnerable taxa will undergo reductions in their distribution area.

## Introduction

During the next century rise of 1–5°C is expected in the global air temperature [1–3]. This increase would be especially significant in Europe, where annual mean air temperatures would be even greater than the global average [4]. Furthermore, according to the Fourth Assessment Report of the IPCC, observed climate trends and future climate projections in Europe show regionally varying changes not only in temperature, but also in rainfall patterns. Thus, a temperature rise throughout Europe is forecast with a marked spike in high-temperature extremes, accompanied by worse meteorological droughts, and more accentuated torrential rains. However, while precipitation in Northern Europe will become heavier, in Southern Europe it will diminish [4]. In this sense, the effects will become more extreme towards the south of Europe, where Mediterranean climatic conditions prevail [5]. This climate zone is characterized by harshly contrasting conditions with wet winters and dry summer [6–8]. In addition, this area, as a "hotspot” of endangered biodiversity, will undergo special impact. [9–11]. Future changes in the magnitude of hydrological drought and its duration show contrasting patterns across Europe, and all these negative aspects are expected to become more severe in Mediterranean peninsulas (Italy, Spain, Italy, Greece, and the Balkans) [12]. Therefore, the Mediterranean area in Southern Europe is of particular relevance for studies examining the effects of global change.

Water temperature is fundamental to the life history of aquatic insects, determining survival, growth, metabolism, phenology and behaviour as well as biotic interactions [13–19]. Air and water temperatures are known to be closely related. Therefore, rising global temperatures are expected to exert a severe impact on freshwater ecosystems. Rising temperatures would be accompanied with diminishing river flow [2,9]. This alteration of the flow regime would change the common pattern of natural hydrologic variation and disturbance, thereby altering habitat dynamics and giving rise to new conditions that would affect especially species with low adaptation capacity [5]. Thus, an altered flow regime would act as ecological bottleneck for aquatic insects [20,21]. Furthermore, these changes would influence species phenology, their distribution range, and the composition and dynamics of communities and are likely to have significant implications for species and habitat conservation [1,22,23]. As a consequence, freshwater habitats are among the most endangered ecosystems in terms of biodiversity loss, because of overexploitation, water pollution, invasive species, flow alteration, and habitat degradation [24,25]. In this sense, watercourses across Europe will be severely threatened by climate change, with far-reaching implications for biological communities [26,27].

In recent years, the high number records of publications on the effect of climate change on freshwater and, therefore, in aquatic macroinvertebrates in recent years reflects the importance of conservation of these ecosystems e.g. [5,28–35]. Species worldwide are dramatically declining due to ongoing climate change accompanied by a reduction of climatically suitable habitats for cold-water aquatic species [29,36]. Freshwater biodiversity has declined faster than either terrestrial or marine biodiversity over the past 30 years and it has been predicted that 15–37% of freshwater species will go extinct due to climate change in the next few decades [37–39]. Species occurring in specific stream zones along the river continuum are expected to respond differentially to climate change due to different thermal regimes [29]. Thus, freshwater biodiversity proves particularly vulnerable to global warming and the sensitivity of aquatic insects depends mainly on: endemicity, preference for springs and cold-water temperatures, emergence period, and feeding requirements [32,35]. Families with a great number of cold-adapted species living at the headwaters would be more vulnerable to global change than families containing a great number of lowland species [35].

Recent studies indicate that the distribution of aquatic macroinvertebrates may be affected by a reduction in habitat for cold-adapted species in high latitudes and elevations, as well as for warm-adapted species at lower latitudes, for habitat specialists and for species with specialized life history traits, such as short emergence periods or narrow ecological niches, e.g. specialized feeding ecologies [29]. Due to the rising water temperature and changing hydrological regime, species from higher elevations may be progressively replaced by generalist species taking advantage of the gradual warming of streams [5,28,40]. Moreover, while river species are expected to shift their distribution upwards in altitude, water warming might additionally facilitate invasions by non-native taxa [5,40,41]. Thus, the relationship between climate and large-scale freshwater assemblages can help us to understand and predict climate-change effects on freshwater ecosystems [6].

Within the “Euro-limpacs” European project, vulnerability was analysed for some aquatic insects groups (Ephemeroptera, Plecoptera, and Trichoptera), presumably very sensitive to temperature change and alterations of river flow due their biology (larvae and nymphs spend the longest period of their cycle within the river) [31,32,42]. These studies have pointed out that the Mediterranean peninsulas of Southern Europe host the largest number of sensitive species [31,35]. In the Sierra Nevada mountain range (southern Spain), two recent studies [5,43] reported that the air temperature increased almost 2°C during the last 50 years accompanied by an increase of 1.63°C on average in water temperature during a 20-year period (1984–2009) affecting macroinvertebrate communities. Caddisfly species richness increased due to global change (increase in air temperature and decrease in discharge) over a 20-year period [5,44]. The results showed that taxa richness was positively related to elevation, with a maximal change at sites of high-intermediate elevation in the study range, where colonizer species have recently been more likely to be detected [5].

To implement the requirements of the European Water Framework Directive (2000/60/ EC; WFD) [45] in Spain, two consecutive national projects were carried out under the acronym GUADALMED (a composed word derived from “Guadal”, an Arabic word for river, and “Med”, from Mediterranean (projects GUADALMED I: HID98-0323-C05-05 and GUADALMED II: REN 2001-3438-C07-06/HID) [46]. Throughout the second project, a predictive model was implemented for the aquatic macroinvertebrates of the Spanish Mediterranean watercourses, called the MEDPACS (MEDiterranean Prediction And Classification System) [47]. This model, based on the RIVPACS/AUSRIVAS predictive approach, involves the use of the Ecological Quality Ratio (EQR) for the number of macroinvertebrate taxa and for two previously developed biotic indices (IBMWP and IASPT, formerly BMWP’ and ASPT’) [47]. The MEDPACS approach has been implemented as a website application available online (http://medpacs.ugr.es). Predictive models of biological communities are based on the use of similarity indices that provide an indication of how a biological community of a particular site is similar to that community elsewhere or, where appropriate, to the reference conditions of a community. In this sense, ratings or biological ordinations of sets of locations can be settled by probabilistic methods (multivariate statistical analysis), and thus the relationship between communities and the possible present disturbances can be established. The goal of working with scenarios is not to predict the future, but to better understand uncertainties in order to make decisions that are robust under a wide range of possible future scenarios [48].

As mentioned above, most published reports are based on climate projections of an increase of 1–5°C on average in the global air temperature that will be accompanied with a decrease in river flow [2,4,9,49]. If changes occur in any environmental condition, organisms have two possible responses: dispersion and colonization of new areas both in latitude and elevation, or, depending on their phenotypic or genotypic plasticity, adaptation to that change [5,27,50–56]. Therefore, in future scenarios of global change, a shift is expected in the distribution range of species that present physiological adaptations to new environmental conditions and dispersive capabilities to new more favourable habitats moving to higher latitudes and elevations [2,57]. The aim of this study is to apply the predictive MEDPACS approach for the analysis of global change in a presumably highly sensitive area, i.e. Mediterranean watercourses. For this purpose, the probability of capturing taxa under current conditions and three possible scenarios of a spatial resolution with accuracy of 500x500 m grids within the Köppen Mediterranean climate zone in Spain and the Balearic Islands have been modelled to detect differences in aquatic macroinvertebrate community. Thus, the starting-point hypothesis is that changes in the local community composition are expected because of either the migration of some taxa seeking favourable new climatic conditions or the extinction in some grids and scenarios of others under unfavourable new conditions.

## Material and Methods

**Fig 1:**
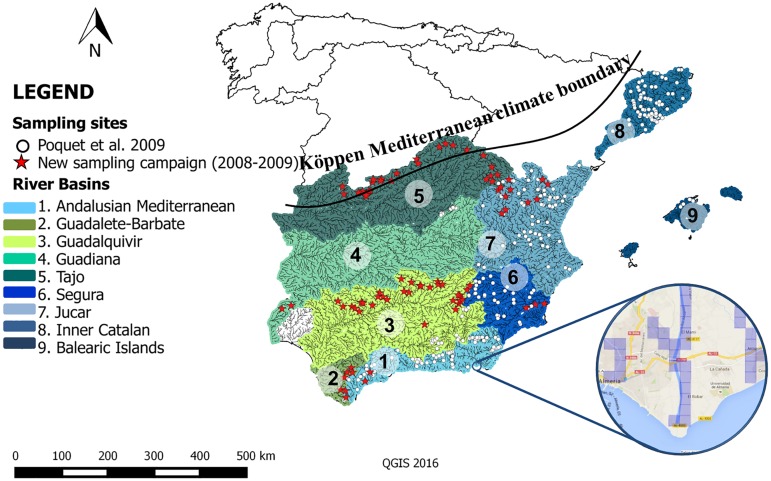
Study area. Black line represents the Köppen (1923) Mediterranean climate boundary. River basins, grids of 500x500 m where model was applied (blue squares in map and zoom), and sampling sites (circles and stars) are represented. “Circles” indicate those sites included in Poquet et al. 2009 and “Stars” those added in the new sampling campaigns.

The study area includes Spain and the Balearic islands within the Köppen Mediterranean climate zone [58]. This represents almost the entire two thirds of the southern Iberian Peninsula (Fig 1). This area is characterized by hot, dry summers, and cold, wet winters with annual temperatures ranging between 42°C and -2°C (mean value of 16–17°C), and annual precipitation ranging from less than 300 mm in the more arid basins of the southeast to over 800 mm in northern basins and in some mountain areas.

As the MEDPACS approach considers temperature and flow, we applied the model to three different global warming scenarios and the associated predicted effects on flow reductions taking into account the ranges predicted in the literature for this area and climate [59]: +1.70°C and -10% flow (scenario 1); +2.45°C and -20% flow (scenario 2); +3.30°C and -30% flow (scenario 3). To increase the applicability of the original MEDPACS model [47], we sampled 85 new sites (in an elevational range from 33 to 1597 m. a.s.l.) between autumn 2008 and 2009 following the same methodology described in the MEDPACS project [47,60] (Fig 1). The area was limited to the Mediterranean arch (along the east coast of Spain) (see Poquet *et al*. 2009 and Fig 1). New sites included headwaters, middle reaches of streams, and the lowlands of major rivers belonging to seven river basins: Andalusian Mediterranean, Guadalete-Barbate, Guadiana, Guadalquivir, Jucar, Segura, and Tajo (Fig 1). To be able to sample in protected areas required permissions were obtained from both the Spanish National and Automous regional environmental authorities.

To verify possible differences in taxa distribution related to global change, the study area was divided into grids of 500 x 500 m, for a total of 151,364 grids containing watercourse stretches within the study area (Fig 1). In the MEDPACS approach, a site was considered outside of the environmental range of the predictive model when it was identified as an outlier by three to five of the best discriminant function (DF) models selected [47]. Once MEDPACS was applied and after the elimination of the sites considered outliers on at least one of the modelled scenarios, a total of 127,640 grids were used for the analyses. MEDPACS calculates the probability of capturing each taxon in all the grids and scenarios with different conditions (considering the current conditions as scenario 0). For the analyses, only taxa that had a capture probability of ≥50% on each grid were considered. Thus, a total of 69 taxa of aquatic macroinvertebrates were included for the predictions.

After modelling expected richness for each grid and scenario, means of taxa richness in different scenarios was calculated and differences between each modelled situation and the current one were explored by univariate ANOVAs with taxa richness as a dependent variable and using scenario and river basin as independent factors. Furthermore, The Ecological Quality Ratio (EQR = observed/expected) was calculated and figured to visualize how the potential climatic changes might affect the Ecological status, following the WFD requirements.

Since changes in EQR suggest differences in macroinvertebrate composition for each grid and scenario, the area of presence and difference between modelled scenarios and scenario 0 were calculated for each taxon were taken as indicative of migration movements. Area of presence (as number of occupied grids with a probability of capture ≥50%) and percentage over the 127,640 included grids were calculated for each taxon in scenario 0 to avoid mistakes of overestimating the effect of global change on some taxa with a small distribution and low probability of capture under current conditions. Differences in the number of occupied grids for each taxon between each future scenario and scenario 0 were calculated, as well as the percentage of change, in order to determine how climate changes would affect each taxon. Altitudinal shifts in taxa ranges were analysed by differences in means (in percentages) using the mean altitude of the taxa in their current distribution and the mean altitude of suitable habitat area under the three scenarios. Thus, we predicted which taxa would be most threatened. Finally, to verify shifts in the distribution of macroinvertebrate taxa related to temperature changes and river flow, the mean altitude at which each taxon would be distributed for each scenario was predicted and compared with the simulated current conditions. Database used for the analyses it is included as a rar compressed Microsoft Access file, divided in four parts because its size (S1–S3 Files)

## Results

**Fig 2:**
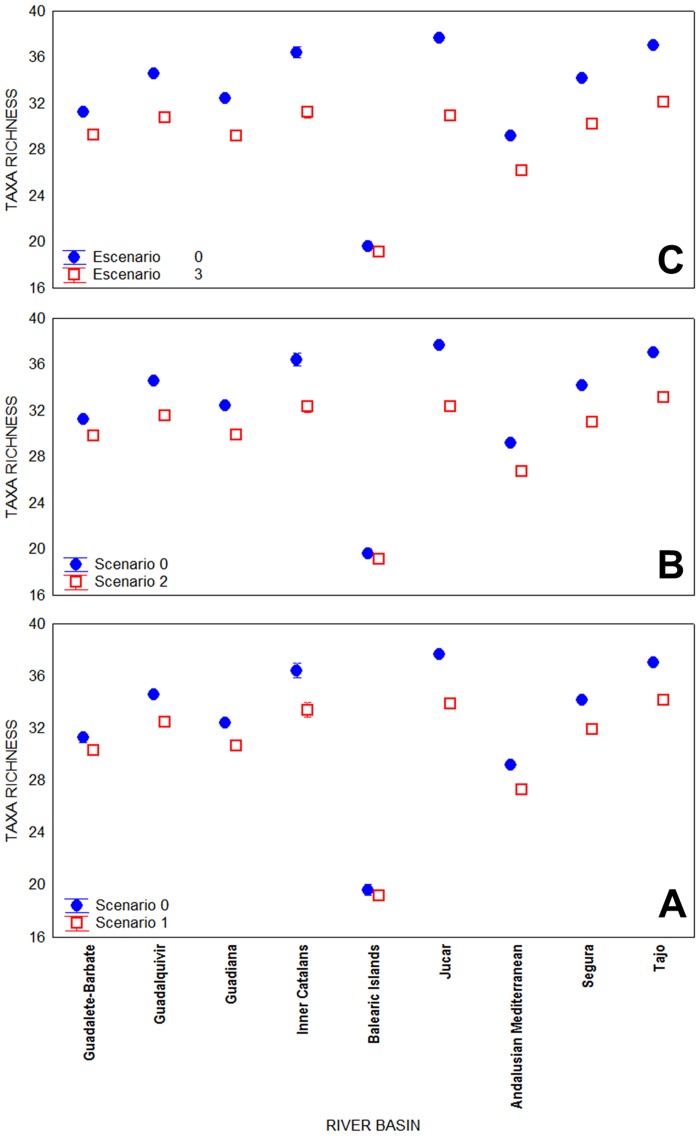
Average (±CI 95%) of taxa richness in each river basin. A: in scenario 0 (current conditions) and scenario 1 (+1.70°C and -10% flow); B: in scenario 0 and scenario 2 (+2.45°C and -20% flow); and C: in scenario 0 and scenario 3 (+3.30°C and -30% flow).

Significant differences in mean taxa richness in each grid within the study area resulted for each future scenarios in comparison with current conditions: scenario 0 [Mean(SE): 34.38(0.03)]; scenario 1 [Mean(SE): 31.98(0.03)]; scenario 2 [Mean(SE): 31.03(0.03)]; scenario 3 [Mean(SE): 30.14(0.02)] (Table 1). Furthermore, the detected decline in taxa richness in different global change scenarios was related to latitudinal distributions of the river basins (Table 1 and Fig 2).

**Table 1:** Summary of the univariate ANOVA on the predicted effectvariations from the starting actual conditions scenario (S0) towards progressive comparative consecutive variation scenarios river basin, and their interactions on taxa richness.

	Type III (SS)	df	F	P
**S1 vs. S0**				
Scenario	68506	1	990.3	0.00
Basin	1604446	8	2899.2	0.00
Scenario*Basin	32607	8	58.9	0.00
**S2 vs. S0**				
Scenario	130704	1	2037.6	0.00
Basin	1460469	8	2845.9	0.00
Scenario*Basin	63143	8	123.0	0.00
**S3 vs. S0**				
Scenario	208346	1	3494.7	0.00
Basin	1316624	8	2760.6	0.00
Scenario*Basin	104025	8	218.1	0.00

**Fig 3:**
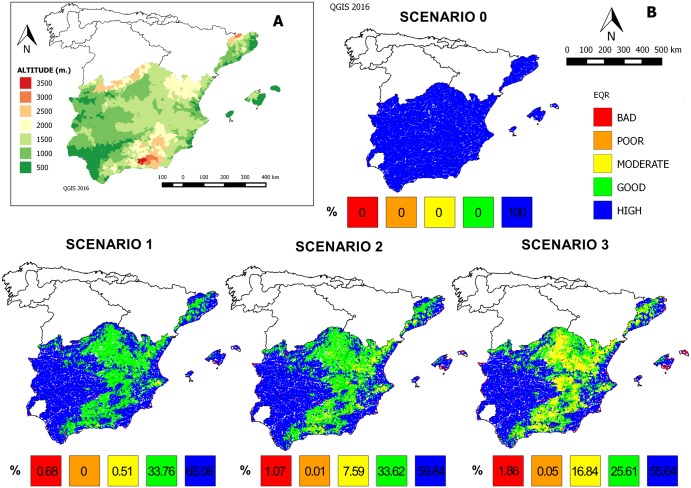
Representation of EQR in the scenarios modelled. A. Altitudinal map of study area. B. Ecological Quality Ratio (EQR) calculated for each grid and scenario: scenario 0 (current conditions), scenario 1 (+1.70°C and -10% flow), scenario 2 (+2.45°C and -20% flow), scenario 3 (+3.30°C and -30% flow). Bottom bars indicate the percentage of grids for each EQR value. Colours are according to the European Water Framework Directive requirements.

According with the WDF, the EQR values vary from almost 1 to 0, indicating “High” or “Bad” ecological status, respectively. Thus, the EQR was calculated on each grid for the three possible scenarios of climate change. As expected, the results showed a decline in EQR throughout the different scenarios (Fig 3B). Transferring the EQR values to Ecological status significances, according with WFD requirements, the EQR in the scenario 0 (simulated current conditions) was estimated as 1 for the total of grids (“High” ecological status) (Fig 3B). The percentage of grids with a value of “High” ecological status (in blue) would be drastically reduced in future modelled scenarios: 65.06% (scenario 1), 59.84% (scenario 2), and 55.64% (scenario 3) (Fig 3B). Thus, throughout different scenarios, the ecological status would change from “High” (blue) to “Good” (green) and finally to “Moderate” (yellow) for most of the grids (see percentages bars in Fig 3). These results indicate a change in the composition of macroinvertebrate taxa in different scenarios of global change in each grid. Furthermore, EQR values indicate that changes would be especially evident in the eastern mountain ranges, where most of the watercourses included in the study grids would change from a “High” in scenario 0 to “Moderate” ecological status in the scenario 3, suggesting evident distributional shifts of macroinvertebrates in the context of global change both in latitude and elevation (Fig 3A).

**Fig 4:**
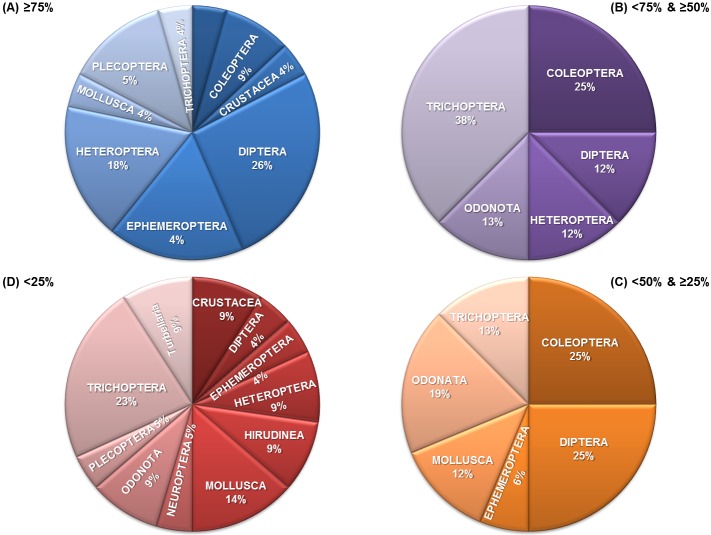
Percentage of taxa for each category of occupied area under current conditions (scenario 0). A (Blue): ≥75%; B (Purple): <75 and ≥ 50%; C (Orange): <50 and ≥25%; D (Red): <25% of grids.

After modelling the probability of capturing taxa at each site and under different scenarios, we calculated the area of presence for individual taxa in scenario 0 or current conditions as percentages of occupied grids with a probability of capture equal to or higher than 50% (Table 2). One-third of the taxa (23 out of 69, 33.33%) would be present in more than 75% of the grids (Table 3; 1 in column 11). These taxa represent almost all the groups (Order/Class) included in the study (Fig 4). Similarly, almost one-third of the taxa (22 out of 69, 31.88%) would be present in an area smaller than 25% of the grids in scenario 0 (Table 3; 4 in column 11), this representing a large proportion of the groups (Fig 4). By contrast, few taxa would be present in less than 50–75% (8 out 69, 11.59%) and 25–50% (16 out 69, 23.19%) of the grids (Table 3; 2 and 3, respectively in column 11).

**Table 2:** List of macroinvertebrate taxa included in the study and their area of presence for each modelled scenario.

1	2	3	4	5	6	7	8	9	10
Taxa	Groups	Grids S0	Grids S1	Grids S2	Grids S3	% S0	% S1	% S2	% S3
**Hydracarina**	Ara	121302	121303	121303	121303	95.03	95.04	95.04	95.04
**Dryopidae**	COL	40351	28004	23111	19036	31.61	21.94	18.11	14.91
**Dytiscidae**	COL	121302	121303	121303	121303	95.03	95.04	95.04	95.04
**Elmidae**	COL	121302	121303	121303	121303	95.03	95.04	95.04	95.04
**Gyrinidae**	COL	33066	20042	15384	11853	25.91	15.70	12.05	9.29
**Haliplidae**	COL	77619	75282	73597	71340	60.81	58.98	57.66	55.89
**Hydraenidae**	COL	77025	70338	66878	62508	60.35	55.11	52.40	48.97
**Hydrophilidae**	COL	54433	43440	39382	34931	42.65	34.03	30.85	27.37
**Scirtidae**	COL	49017	35214	28280	21658	38.40	27.59	22.16	16.97
**Atyidae**	CRU	1	0	0	0	0.00	0.00	0.00	0.00
**Gammaridae**	CRU	3216	3538	3622	3668	2.52	2.77	2.84	2.87
**Ostracoda**	CRU	119666	120270	120465	120633	93.75	94.23	94.38	94.51
**Athericidae**	DIP	32439	20275	16184	12930	25.41	15.88	12.68	10.13
**Ceratopogonidae**	DIP	120338	120287	120280	120266	94.28	94.24	94.23	94.22
**Chironomidae**	DIP	121302	121303	121303	121303	95.03	95.04	95.04	95.04
**Culicidae**	DIP	13734	6292	4059	2272	10.76	4.93	3.18	1.78
**Dixidae**	DIP	121302	121303	121303	121303	95.03	95.04	95.04	95.04
**Empididae**	DIP	46540	35039	29992	25849	36.46	27.45	23.50	20.25
**Limoniidae**	DIP	121302	121303	121303	121303	95.03	95.04	95.04	95.04
**Psychodidae**	DIP	30326	18997	15580	12487	23.76	14.88	12.21	9.78
**Simuliidae**	DIP	121302	121303	121303	121303	95.03	95.04	95.04	95.04
**Stratiomyidae**	DIP	45549	33228	28680	24178	35.69	26.03	22.47	18.94
**Tabanidae**	DIP	117154	116879	116804	116720	91.78	91.57	91.51	91.44
**Tipulidae**	DIP	70774	61627	56347	50552	55.45	48.28	44.15	39.61
**Baetidae**	EPH	121302	121303	121303	121303	95.03	95.04	95.04	95.04
**Caenidae**	EPH	119612	120049	120189	120339	93.71	94.05	94.16	94.28
**Ephemerellidae**	EPH	95315	91614	89550	87519	74.67	71.78	70.16	68.57
**Ephemeridae**	EPH	17245	9533	6948	4850	13.51	7.47	5.44	3.80
**Heptageniidae**	EPH	47938	34783	29839	25012	37.56	27.25	23.38	19.60
**Leptophlebiidae**	EPH	110744	108926	108218	107687	86.76	85.34	84.78	84.37
**Corixidae**	HET	88830	92472	93988	95397	69.59	72.45	73.64	74.74
**Gerridae**	HET	120161	119919	119768	119667	94.14	93.95	93.83	93.75
**Hydrometridae**	HET	103470	100909	100116	99474	81.06	79.06	78.44	77.93
**Naucoridae**	HET	3179	3580	3913	4162	2.49	2.80	3.07	3.26
**Nepidae**	HET	1402	1296	1299	1196	1.10	1.02	1.02	0.94
**Notonectidae**	HET	113735	114775	115255	115692	89.11	89.92	90.30	90.64
**Veliidae**	HET	98865	93179	90841	88132	77.46	73.00	71.17	69.05
**Erpobdellidae**	HIR	1	0	0	0	0.00	0.00	0.00	0.00
**Glossiphoniidae**	HIR	1	0	0	0	0.00	0.00	0.00	0.00
**Sialidae**	NEU	155	21	0	0	0.12	0.02	0.00	0.00
**Ancylidae**	MOL	105267	107613	108442	109019	82.47	84.31	84.96	85.41
**Hydrobiidae**	MOL	30685	20574	16683	13559	24.04	16.12	13.07	10.62
**Lymnaeidae**	MOL	27028	15422	11330	7744	21.18	12.08	8.88	6.07
**Physidae**	MOL	1230	1404	1585	1699	0.96	1.10	1.24	1.33
**Planorbidae**	MOL	241	214	172	108	0.19	0.17	0.13	0.08
**Sphaeriidae**	MOL	31579	19764	15544	12256	24.74	15.48	12.18	9.60
**Aeshnidae**	ODO	42513	29837	24868	20206	33.31	23.38	19.48	15.83
**Calopterygidae**	ODO	21920	12624	9505	6816	17.17	9.89	7.45	5.34
**Coenagrionidae**	ODO	72397	80497	83324	85973	56.72	63.07	65.28	67.36
**Cordulegasteridae**	ODO	29496	17723	13729	10685	23.11	13.89	10.76	8.37
**Gomphidae**	ODO	56138	44792	39343	34141	43.98	35.09	30.82	26.75
**Libellulidae**	ODO	56642	66391	70934	75087	44.38	52.01	55.57	58.83
**Leuctridae**	PLE	112889	111732	110878	109827	88.44	87.54	86.87	86.04
**Nemouridae**	PLE	108002	106469	105996	105745	84.61	83.41	83.04	82.85
**Perlidae**	PLE	8112	4625	3766	3045	6.36	3.62	2.95	2.39
**Perlodidae**	PLE	101822	101119	101017	101031	79.77	79.22	79.14	79.15
**Brachycentridae**	TRI	817	585	495	410	0.64	0.46	0.39	0.32
**Glossosomatidae**	TRI	12561	6835	4753	2890	9.84	5.35	3.72	2.26
**Hydropsychidae**	TRI	92623	88136	86340	85030	72.57	69.05	67.64	66.62
**Hydroptilidae**	TRI	81858	75691	70895	65359	64.13	59.30	55.54	51.21
**Leptoceridae**	TRI	86805	80015	76040	71642	68.01	62.69	59.57	56.13
**Limnephilidae**	TRI	67538	58597	53984	49448	52.91	45.91	42.29	38.74
**Philopotamidae**	TRI	9783	7030	5980	4929	7.66	5.51	4.69	3.86
**Polycentropodidae**	TRI	52042	37873	31762	25820	40.77	29.67	24.88	20.23
**Psychomyiidae**	TRI	10820	6396	4563	3298	8.48	5.01	3.57	2.58
**Rhyacophilidae**	TRI	43324	29555	24577	20571	33.94	23.15	19.25	16.12
**Sericostomatidae**	TRI	20081	12477	9739	7401	15.73	9.78	7.63	5.80
**Dugesiidae**	Tur	9625	4770	2884	1463	7.54	3.74	2.26	1.15
**Planariidae**	Tur	1492	1013	859	753	1.17	0.79	0.67	0.59

Groups (ORDER or Class); Ara: Arachnida; COL: Coleoptera; CRU: Crustacea; DIP: Diptera; EPH: Ephemeroptera; HET: Heteroptera; HIR: Hirudinea; NEU: Neuroptera; MOL: Mollusca; ODO: Odonata; PLE: Plecoptera; TRI: Trichoptera; Tur: Turbellaria).

Columns 3–6: Number of grids where each taxon is present with a probability of capture ≥50% for each scenario. S0 to S3: Scenario 0 (current conditions), scenario 1 (+1.70°C and -10% flow), scenario 2 (+2.45°C and -20% flow), scenario 3 (+3.30°C and -30% flow).

Columns 7–9: Percentages of occupied grids for each taxon with respect to the total of include grids in each scenario. S0 to S3: Scenario 0 (current conditions), scenario 1 (+1.70°C and -10% flow), scenario 2 (+2.45°C and -20% flow), scenario 3 (+3.30°C and -30% flow).

**Table 3:** Distributional and altitudinal shifts between different modelled scenario and current situation (scenario 0) for each taxon.

1	2	3	4	5	6	7	8	9	10	11
Taxa	Group	IBMWP score	DIF_%S1-S0	DIF_%S2-S0	DIF_%S3-S0	MeanAlt S0	DIF_AltS1-S0	DIF_Alt S2-S0	DIF_Alt S3-S0	AP S1
**Gammaridae**	CRU	6	10.01	12.62	14.05	81.44	28.57	37.03	40.18	**4**
**Atyidae**	CRU	6	-100.00	-100.00	-100.00	100.00				**4**
**Erpobdellidae**	HIR	3	-100.00	-100.00	-100.00	100.00				**4**
**Glossiphoniidae**	HIR	3	-100.00	-100.00	-100.00	100.00				**4**
**Planorbidae**	MOL	3	-11.20	-28.63	-55.19	144.81	-14.44	-55.28	-115.18	**4**
**Libellulidae**	ODO	8	17.21	25.23	32.56	353.89	29.05	42.07	53.69	**3**
**Coenagrionidae**	ODO	6	11.19	15.09	18.75	390.85	26.16	34.16	41.93	**2**
**Physidae**	MOL	3	14.15	28.86	38.13	397.40	31.66	37.43	45.80	**4**
**Corixidae**	HET	3	4.10	5.81	7.39	416.86	10.65	15.12	19.30	**2**
**Naucoridae**	HET	3	12.61	23.09	30.92	460.21	36.92	54.49	67.42	**4**
**Notonectidae**	HET	3	0.91	1.34	1.72	477.00	1.45	1.65	2.01	**1**
**Caenidae**	EPH	4	0.37	0.48	0.61	489.26	1.96	2.63	3.38	**1**
**Gerridae**	HET	3	-0.20	-0.33	-0.41	491.75	-0.75	-1.31	-1.72	**1**
**Ostracoda**	CRU	3	0.50	0.67	0.81	495.78	1.46	1.89	2.32	**1**
**Ancylidae**	MOL	6	2.23	3.02	3.56	498.24	-1.14	-1.53	-1.57	**1**
**Hydracarina**	Ara	4	0.00	0.00	0.00	499.16	0.00	0.00	0.00	**1**
**Dytiscidae**	COL	3	0.00	0.00	0.00	499.16	0.00	0.00	0.00	**1**
**Elmidae**	COL	5	0.00	0.00	0.00	499.16	0.00	0.00	0.00	**1**
**Chironomidae**	DIP	2	0.00	0.00	0.00	499.16	0.00	0.00	0.00	**1**
**Dixidae**	DIP	4	0.00	0.00	0.00	499.16	0.00	0.00	0.00	**1**
**Limoniidae**	DIP	4	0.00	0.00	0.00	499.16	0.00	0.00	0.00	**1**
**Simuliidae**	DIP	5	0.00	0.00	0.00	499.16	0.00	0.00	0.00	**1**
**Baetidae**	EPH	4	0.00	0.00	0.00	499.16	0.00	0.00	0.00	**1**
**Ceratopogonidae**	DIP	4	-0.04	-0.05	-0.06	502.79	0.17	0.20	0.24	**1**
**Tabanidae**	DIP	4	-0.23	-0.30	-0.37	504.68	2.07	2.68	3.42	**1**
**Hydroptilidae**	TRI	6	-7.53	-13.39	-20.16	508.37	-15.61	-22.85	-32.88	**2**
**Nepidae**	HET	3	-7.56	-7.35	-14.69	514.41	33.97	45.87	43.20	**4**
**Leuctridae**	PLE	10	-1.02	-1.78	-2.71	516.92	-1.63	-1.86	-1.93	**1**
**Hydrometridae**	HET	3	-2.48	-3.24	-3.86	517.77	-1.20	-3.28	-5.99	**1**
**Leptophlebiidae**	EPH	10	-1.64	-2.28	-2.76	525.82	-0.59	-1.47	-2.34	**1**
**Nemouridae**	PLE	7	-1.42	-1.86	-2.09	530.92	-1.37	-2.66	-3.76	**1**
**Perlodidae**	PLE	10	-0.69	-0.79	-0.78	535.40	-3.69	-5.98	-8.36	**1**
**Ephemerellidae**	EPH	7	-3.88	-6.05	-8.18	536.22	-7.54	-11.16	-14.05	**1**
**Haliplidae**	COL	4	-3.01	-5.18	-8.09	542.12	-15.24	-26.44	-40.19	**2**
**Hydropsychidae**	TRI	5	-4.84	-6.78	-8.20	542.52	7.67	10.93	12.91	**1**
**Leptoceridae**	TRI	10	-7.82	-12.40	-17.47	546.24	-16.55	-26.76	-39.90	**2**
**Veliidae**	HET	3	-5.75	-8.12	-10.86	551.69	-6.19	-12.91	-21.65	**1**
**Hydraenidae**	COL	5	-8.68	-13.17	-18.85	572.96	-3.29	-6.01	-12.10	**2**
**Tipulidae**	DIP	5	-12.92	-20.38	-28.57	590.21	-1.39	-4.27	-10.29	**2**
**Hydrophilidae**	COL	3	-20.20	-27.65	-35.83	633.36	17.33	17.75	12.74	**3**
**Limnephilidae**	TRI	7	-13.24	-20.07	-26.78	645.69	37.17	53.17	66.92	**2**
**Gomphidae**	ODO	8	-20.21	-29.92	-39.18	656.71	32.82	51.09	67.10	**3**
**Polycentropodidae**	TRI	7	-27.23	-38.97	-50.39	712.96	54.41	81.33	107.61	**3**
**Stratiomyidae**	DIP	4	-27.05	-37.03	-46.92	717.23	57.08	76.11	93.11	**3**
**Aeshnidae**	ODO	8	-29.82	-41.50	-52.47	726.98	50.93	67.13	80.65	**3**
**Scirtidae**	COL	3	-28.16	-42.31	-55.82	738.59	56.80	95.66	144.04	**3**
**Rhyacophilidae**	TRI	7	-31.78	-43.27	-52.52	741.93	48.50	67.44	81.98	**3**
**Empididae**	DIP	4	-24.71	-35.56	-44.46	745.71	51.08	74.77	95.78	**3**
**Dryopidae**	COL	5	-30.60	-42.73	-52.82	762.18	54.39	71.46	80.04	**3**
**Hydrobiidae**	MOL	3	-32.95	-45.63	-55.81	764.33	37.37	46.90	49.31	**3**
**Heptageniidae**	EPH	10	-27.44	-37.76	-47.82	774.34	74.38	107.69	139.67	**3**
**Athericidae**	DIP	10	-37.50	-50.11	-60.14	799.05	69.05	92.52	119.37	**3**
**Lymnaeidae**	MOL	3	-42.94	-58.08	-71.35	802.20	84.97	129.22	174.70	**4**
**Psychomyiidae**	TRI	8	-40.89	-57.83	-69.52	802.29	-68.04	-106.41	-155.38	**4**
**Gyrinidae**	COL	3	-39.39	-53.47	-64.15	805.56	79.81	111.37	137.59	**3**
**Philopotamidae**	TRI	8	-28.14	-38.87	-49.62	809.04	8.25	27.08	56.05	**4**
**Sphaeriidae**	MOL	3	-37.41	-50.78	-61.19	832.39	73.16	101.42	130.93	**3**
**Cordulegasteridae**	ODO	8	-39.91	-53.45	-63.77	835.03	83.43	114.16	137.42	**4**
**Calopterygidae**	ODO	8	-42.41	-56.64	-68.91	842.59	68.29	102.06	137.37	**4**
**Psychodidae**	DIP	4	-37.36	-48.62	-58.82	859.99	73.97	100.02	127.57	**3**
**Ephemeridae**	EPH	10	-44.72	-59.71	-71.88	919.81	71.48	106.73	140.44	**4**
**Sericostomatidae**	TRI	10	-37.87	-51.50	-63.14	929.36	61.85	88.15	111.01	**4**
**Culicidae**	DIP	2	-54.19	-70.45	-83.46	930.84	101.89	147.41	164.59	**4**
**Dugesiidae**	Tur	5	-50.44	-70.04	-84.80	938.03	45.85	31.25	-37.75	**4**
**Perlidae**	PLE	10	-42.99	-53.57	-62.46	982.89	11.08	17.96	23.65	**4**
**Sialidae**	NEU	4	-86.45	-100.00	-100.00	993.55	-55.45			**4**
**Glossosomatidae**	TRI	8	-45.59	-62.16	-76.99	997.88	86.77	133.63	174.16	**4**
**Planariidae**	Tur	5	-32.10	-42.43	-49.53	1199.73	81.22	117.38	143.30	**4**
**Brachycentridae**	TRI	10	-28.40	-39.41	-49.82	1333.90	85.75	124.48	162.92	**4**

Taxa are listed according with the mean altitude in scenario 0 (column 7).

Groups (ORDER or Class); Ara: Arachnida; COL: Coleoptera; CRU: Crustacea; DIP: Diptera; EPH: Ephemeroptera; HET: Heteroptera; HIR: Hirudinea; NEU: Neuroptera; MOL: Mollusca; ODO: Odonata; PLE: Plecoptera; TRI: Trichoptera; Tur: Turbellaria).

Columns 4–6: Percentages of shifts in number of grids where each taxa is present between each scenario modelled and the scenario 0 respectively. Negative values represent reduction of area of presence and positive values represent enlargement of area of presence.

Columns 7: Mean altitude (m) for each taxa on scenario 0.

Columns 8–10: Shift in mean altitudinal distribution range between each modelling scenario and the scenario 0 respectively. Negative values represent downstream movements and positive values represent upstream movements.

Column 11: Category of the area of presence (AP) for each taxon in scenario 0; 1: ≥75%; 2: <75 and ≥50%; 3: <50 and ≥25%; 4: <25% of grids occupied.

**Fig 5:**
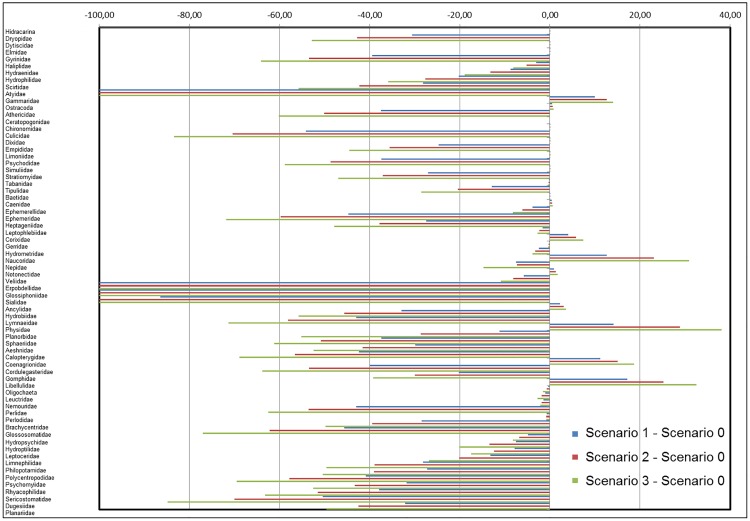
Percentage of shifts in area of presence for each taxon. Difference in number of grids where taxa are present with probability of capture ≥50% between each scenario modelled: scenario 1 (+1.70°C and -10% flow), scenario 2 (+2.45°C and -20% flow), and scenario 3 (+3.30°C and -30% flow), and scenario 0 (current conditions) respectively.

In relation to shifts in the number of occupied grids, i.e. their area of presence, few taxa (8 out 69, 11.59%): Hydracarina (Arachnidae) Dytiscidae and Elmidae (Coleoptera), Chironomidae, Dixidae, Limoniidae, and Simuliidae (Diptera), and Baetidae (Ephemeroptera), would maintain the same area of presence for all the modelled future scenarios and with current conditions in scenario 0 (Fig 5 and Table 3; columns 4–6, percentage of change = 0%). Nevertheless, most of the taxa would show changes between the number of grids occupied in each modelled scenario and the number of grids occupied in scenario 0 (Table 3; columns 4–6, respectively), in all cases with a probability of capture ≥50%. Thus, many of them (47 out of 69, 68.12%) belonging to Coleoptera, Crustacea, Diptera, Ephemeroptera, Heteroptera, Hirudinea, Mollusca, Neuroptera, Odonata, Plecoptera, Trichoptera, and Turbellaria would reduce their area of presence (Table 2), showing a negative percentage of change between the number of grids occupied in each modelled scenario and the number of grids occupied in scenario 0 (Fig 5 and Table 3; columns 4–6, negative percentage of change). Nevertheless, it bears highlighting that some of these taxa—Haliplidae and Hydraenidae (Coleoptera), Ceratopogonidae and Tabanidae (Diptera), Ephemerellidae and Leptophlebiidae (Ephemeroptera), Gerridae, Hydrometridae, Nepidae, and Veliidae (Heteroptera), Leuctridae, Nemouridae, and Perlodidae (Plecoptera), Hydropsychidae and Leptoceridae (Trichoptera)—would have a final percentage shift lower than 20% in scenario 3 and must be carefully considered as a trend of change. In addition, only 4 taxa (5.80%), i.e. Atyidae (Crustacea), Erpobdellidae and Glossiphoniidae (Hirudinea), and Sialidae (Neuroptera), would not be captured in modelled future scenarios with a probability ≥50%, as occurs in scenario 0 (Fig 5 and Table 3; columns 4–6). By contrast, only 10 taxa (14.49%) belonging to Crustacea, Ephemeroptera, Heteroptera, Mollusca, and Odonata would show an enlargement of their area of presence (Table 2), suggesting a favourable effect of global change (Fig 5 and Table 3; columns 4–6, positive percentages of change). Remarkably, only 3 taxa, namely Naucoridae (Heteroptera), Physidae (Mollusca), and Libellulidae (Odonata), would show a positive change higher than 20% in their area of presence from being benefited by global change.

Comparing taxa strategies within groups, we found that in 7 cases (Coleoptera, Diptera, Hirudinea, Neuroptera, Plecoptera, Trichoptera, and Turbellaria), all of the taxa would undergo a negative shift (reduction) in their area of presence under the climate conditions modelled (Table 3; negative percentages of change in columns 4–6). On the contrary, in 5 groups (Crustacea, Ephemeroptera, Heteroptera, Mollusca, and Odonata), the shifts would be positive or negative depending on the taxa (Table 3; positive or negative percentages of change in columns 4–6).

**Fig 6:**
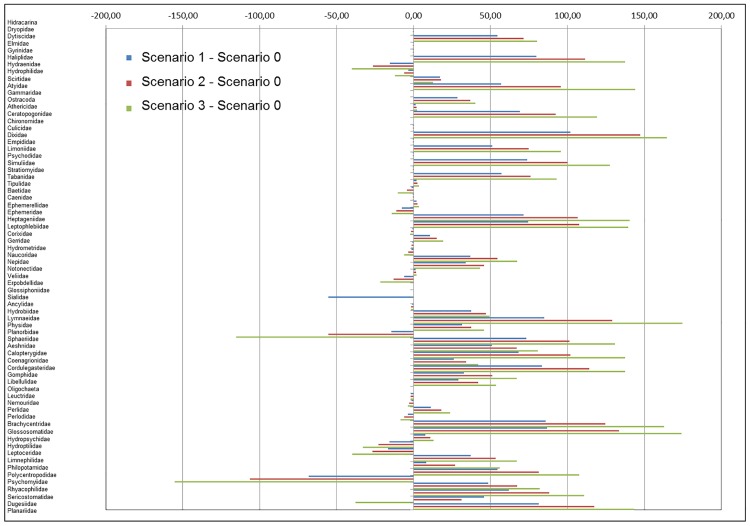
Shifts in altitudinal distribution range for each taxon. Difference in altitudinal distribution range (mean altitude) of taxa present with a probability of capture ≥50%, between each scenario modelled: scenario 1 (+1.70°C and -10% flow), scenario 2 (+2.45°C and -20% flow), and scenario 3 (+3.30°C and -30% flow), and scenario 0 (current conditions) respectively.

In terms of the altitudinal distribution range of taxa in each of the scenarios modelled (Fig 6 and Table 3: columns 8–10), most of them (40 out of 69; 57.97%) belonging to Coleoptera, Crustacea, Diptera, Ephemeroptera, Heteroptera, Mollusca, Odonata, Plecoptera, Trichoptera, and Turbellaria would change their distribution range under modelled future conditions, moving upstream from lower to higher elevations (Fig 6 and Table 3; columns 8–10, positives values). Some of these changes would involve only a few meters (less than 50 m) in many taxa: Hydrophilidae (Coleoptera); Gammaridae and Ostracoda (Crustacea); Ceratopogonidae and Tabanidae (Diptera); Caenidae (Ephemeroptera); Corixidae, Nepidae, and Notonectidae (Heteroptera); Hydrobiidae (Mollusca); Coenagrionidae (Odonata); Perlidae (Plecoptera); and Hydropsychidae (Trichoptera). By contrast, a few taxa (16 out 69, 23.19%) belonging to Coleoptera, Diptera, Ephemeroptera, Heteroptera, Mollusca, Neuroptera, Plecoptera, and Trichoptera would move downstream (Fig 6 and Table 3; columns 8–10, negative values). It bears indicating that a negative shift of less than 50 meters was predicted for taxa such as: Gerridae, Haliplidae, and Hydraenidae (Coleoptera); Tipulidae (Diptera); Ephemerellidae and Leptophebiidae (Ephemeroptera); Hydrometridae and Veliidae (Heteroptera); Ancylidae (Mollusca); Leuctridae, Nemouridae, and Perlodidae (Plecoptera); Hydroptilidae and Leptoceridae (Trichoptera); and Dugesiidae (Turbellaria). With respect to the 4 taxa that would not have a probability of capture ≥50% in the modelled scenarios, only one, Sialidae (Neuroptera), would disappear in scenarios 2 and 3, while in scenario 1 this taxon would move downstream (Table 3; columns 8–10). Furthermore, one taxon (1 out 69; 1.45%), Dugesiidae (Turbellaria), would move upstream in scenarios 1 and 2, but in scenario 3 would drastically switch strategies and move to lower elevations (Table 3). Finally, eight taxa (8 out 69; 11.59%) would maintain the same area of presence (Arachnidae, Dytiscidae, Elmidae, Chironomidae, Dixidae, Limoniidae, Simuliidae, and Baetidae) as well as the same altitudinal distribution range (Fig 6 and Table 3; columns 8–10, values = 0).

As done with percentage of change in occupied grids for different scenarios, comparisons of taxa within each group in relation to the altitudinal distribution range were analysed and differences were also detected. In most of the groups,—Crustacea, Heteroptera, Hirudinea, Neuroptera, Mollusca, Odonata, Plecoptera, Trichoptera, and Turbellaria—all of their taxa would undergo shifts in altitude (Table 3; columns 8–10), but up- or downstream depending of taxa (Table 3; columns 8–10, positive or negative values). By contrast, for Odonata and Turbellaria, all of their taxa would move upstream (Table 3; columns 8–10) whereas for Hirudinea and Neuroptera, all of their taxa would move downstream (Table 3; columns 8–10).

Furthermore, most of the taxa that would reduce their area of presence as a lower number of grids where they would be present with high probabilities (Table 3; columns 4–6, negative percentages; 31 out 47, 65.96%) would also move upwards in elevation (Table 3; columns 8–10, positive values). By contrast, only 15 of them (15 out 47, 31.91%) would move downwards in mean altitude (Table 3; columns 11–13, negative values). And only one (2.13%), Dugesiidae (Turbellaria), would move upstream both in scenario 1 and 2, but would descend in elevation in scenario 3. On the other hand, most of the taxa that would enlarge their area of presence (Table 3; columns 4–6, positive percentage; 9 out 10, 90%) would also ascend in elevation (Table 3; columns 8–10, positive values) and only one of them (10%) would descend (Table 3; columns 8–10, negative values) in the modelled scenarios.

In relation to the river basins where taxa could live under different scenarios, we observed that south-western river basins (Andalusian Mediterranean, Guadalete-Barbate, and Guadiana) would be especially affected. On the contrary, in the Balearic Islands there was a predicted trend to increase the presence of some taxa. Thus taxa predicted under current conditions to have a probability of capture ≥50%, such as Atyidae, Culicidae, Erpobdellidae, and Glossiphonidae, were present only in the Andalusian Mediterranean basin. Other taxa would not be captured with a probability ≥50% in one, two or the three of the scenarios in: the Guadalete-Barbate (Glossosomatidae), in the Guadiana (Culicidae, Perlidae, Philopotamidae, Psychomiidae or Sericostomatidae), or in both basins (Dugesiidae or Ephemeridae). In other basins, such as the Andalusian Mediterranean, Jucar, Guadalquivir, and inner Catalonia, a few taxa would disappear in all the modelled scenarios: Nepidae (Andalusian Mediterranean), Planariidae (Jucar), Sialidae (Jucar and Guadalquivir), and Planorbidae (Inner Catalonia). In addition, other taxa would not be captured with a probability ≥50% in the Tajo river basin in different scenarios (Nepidae, Planorbidae or Sialidae), while another taxon, Physidae, would be present with a probability of capture ≥50% for the first time in this basin in modelled future scenarios. Finally, in the river basins of the Balearic Islands, several families would be benefited by simulated new climatic conditions, such as Hydroptilidae, Leptophlebiidae, Nemouridae or Perlodidae. And only one taxon, Rhyacophilidae, would disappear from this basin in future modelled scenarios.

## Discussion

After the capture probability of 69 aquatic macroinvertebrates was modelled under different scenarios of global change in the Mediterranean study area, our results indicate that the community would be drastically affected by the projected water-temperature rises and subsequent reduction in river flow. Altough the taxa list considered would be similar in each scenario for the entire study area (only 4 taxa would not have a probability of capture ≥50% in future modelled scenarios), their distribution pattern was predicted to vary under all the new climate conditions in relation to latitude and elevation. Families as Ephemeridae, Perlidae, Sericostomatidae, and Brachycentridae among others (31,32,35,42), with large numbers of vulnearable species could not adapt to different scenarios, showing a contraction in their distribution area, implying the disappearance of most of the sites where, under current conditions, the probability of capture was ≥50%.

The calculated EQR values suggest differences in the ecological status of sites for each scenario. Changes in EQR values would prove especially evident in the eastern mountain ranges (Fig 3A), highlighting the effect of elevation on the distributional shifts of aquatic macroinvertebrates (Fig 3) and the vulnerability of this area against future global changes. Fig 3 clearly illustrates how the EQR values would decrease and therefore the ecological status of the river would worsen over the different scenarios in relation to the elevational ranges of the eastern mountains (Fig 3A). Consequently, the communities are predicted to change their distribution range by moving to intermediate-high elevations (1500–2500 m. a.s.l.; Fig 3A, Table 3). In this sense, although future projections of global change indicate a reduction of richness worldwide, at regional or local scales, number of species could also increase [23,61,62]. In accordance with these expectations, even considering the limitations of the identification level of this study, our results show that in a large area such as the Mediterranean part of the Spanish Iberian Peninsula and the Balearic Islands, the taxa composition would be similar under future conditions but the average richness in each local grids would be significantly reduced in different scenarios (Fig 2). Thus, changes in the local community composition are expected, either because of migration of some taxa as an adaptation strategy to new climate conditions, or extinction of other taxa in some areas and scenarios. Facing the possible changes in environmental conditions, organisms have two possible responses: dispersion and colonization of new areas; or, depending on their phenotypic or genotypic plasticity, adaptation to that change [27]. Thus, according to the dispersion strategy, latitudinal and altitudinal migration in association with climate change has been studied in many species [5,27,50–56]. Therefore, a shift is expected in the distribution range of species that present physiological adaptations to new environmental conditions and dispersive capabilities to new, more favourable habitats by moving to higher latitudes and altitudes [2,57].

As expected, most of the taxa including in the present study (68.12%) would reduce the number of sites occupied in different scenarios, i.e. their area of presence, due to global change. Few of them, those with a high probability of capture and large occupation area under current conditions (scenario 0) as Perlodidae (Plecoptera), Leptophlebidae (Ephemeroptera), Ceratopogonidae and Tabanidae (Diptera), and Gerridae (Heteroptera) among others, would have a very low shift in percentage of occupied area (lower than 10%). Nevertheless, a large proportion of taxa (25 out 47, 53.19%) would present greater habitat losses, disappearing in even more than the 50% of grids in which they are present under the simulated current conditions. It is remarkable that most of these taxa have a small area of presence (lower than 50%) in scenario 0, such as Sphaeriidae (Mollusca), Cordulegasteridae (Odonata), Cullicidae (Diptera), Ephemeridae (Ephemeroptera), Sericostomatidae (Trichoptera) or Dugesiidae (Turbellaria). In addition, all these taxa have different IBMWP scores [63,64], ranging between 3 and 10 (Table 3; column 3), highlighting the vulnerability of these Mediterranean ecosystems (in high and low elevations) to global change. Not only sensitive taxa with a high score in the biological index IBMWP would be affected by new climatic conditions, but also families which would presumably be better adapted to changes in their habitat will be threatened. Only a few taxa with a low IBMWP score and occupying a small percentage of grids in scenario 0, would have a poor probability of capture < 50% in the modelled scenarios, such as Atyidae (Crustacea), Erpobdellidae and Glossiphoniidae (Hirudinea), and Sialidae (Neuroptera). On the contrary, a small percentage of taxa (26.08%) would be benefitted by rising temperatures and would enlarge their area of presence or would maintain the same distribution range, as Hydracarina (Arachnida); Dytiscidae and Elmidae (Coleoptera); Chironomidae, Dixidae, Limoniidae, and Simuliidae (Diptera); and Baetidae (Ephemeroptera). In this case, the taxa with a largest increase in occupation within the modelled scenarios would have a small area of presence (lower than 50%) in scenario 0. On the other hand, taxa that would have a null or small increase in occupation area in the modelled scenarios would have a large area of presence (larger than 50%) in scenario 0. Most of these taxa, such as Chironomidae (Diptera), Dytiscidae (Coleoptera), Caenidae (Ephemeroptera), Elmidae (Coleoptera), and Coenagrionidae (Odonata), have a low or medium IBMWP score (between 2 and 6), suggesting a low vulnerability to new conditions.

As several studies on freshwater species have shown, shifts in area of presence of taxa would be accompanied by a shift in latitude and altitudinal distributional ranges (both positive and negative) in response to climate warming and other factors [2]. Most of the taxa would show very small elevational changes (<50 m) either up or downstream. These taxa would occupy a large proportion of grids under current conditions (scenario 0) and have a low average altitudinal distribution range (500–600 m a.s.l.), suggesting a high dispersion capacity and adaptation to inhabit lowlands with warmer water, such as Diptera, Heteroptera, Coleoptera, and Odonata. Meanwhile, taxa with a probability of capture ≥50% that would occupy an area less than 50% of the grids in the scenario 0 and that on average occupy a high altitudinal distribution range (1000–1500 m a.s.l.), having limited dispersion capacity and narrow ecological requirements, would undergo the greatest elevational shifts (>100 m), such as Lymnaeidae, Ephemeridae, Culicidae, Glossosomatidae or Planariidae among others.

Finally, our results indicate that the strategies that the different taxa would follow to face rising temperatures and the subsequent lower of flow, are consistent with previous studies in the global-change context [65]. Thus, most of the taxa (65.96%) would be affected by global change not only by reducing their area of presence, but also by rising in elevation to colonize new sites. In this case, taxa climbing in elevation are those having a small area of presence under current conditions (scenario 0) and ranging in average elevation between 700 and 1500 m. a.s.l. The fact that these taxa inhabit mid-high elevations in scenario 0 and therefore temperate to cold waters, suggests an appropriate capacity of dispersion to higher elevations seeking lower water temperatures. Meanwhile, some taxa (31.91%) distributed at medium elevations (500–600 m a.s.l.) in current conditions would reduce their area of presence in a very low percentage and moving downstream only few meters (most of them less than 100 m.). Warm water tolerant taxa as Planorbiidae, Hydroptilidae, Haliplidae, Psychodidae, or Dugesiidae, with high capacity of living in a very diverse range of habitats, when grids with suitable conditions would increase, and non-warm temperature tolerant competitive taxa would move upwards reducing negative effect of the ecological competence. It is notable that in Ephemeroptera, Plecoptera, and Trichoptera (EPT), almost all of the taxa would follow both strategies. Most Ephemeroptera, except Baetidae and Caenidae, would be progressively affected by reducing their distribution area moving upwards. Most Plecoptera would lose distribution area but at a very low percentage (<10%) maintaining almost the same average on altitudinal range in future scenarios, while only one family (Perlidae) would have a loss of greater than 50%, accompanied by movement upstream of few meters (<50 m.). Finally, most of Trichoptera taxa would reduce their habitat but move to higher elevations. Meanwhile, most of the taxa distributed in an altitudinal range lower than 500 m a.s.l., belonging to the orders Coleoptera, Diptera, Heteroptera or Odonata (mostly OCH) as well as crustaceans and molluscs, would benefit from global warming by expanding their area of presence in addition to positively shifting in elevation. In this sense, before a temperature rise and changes in hydrological regimes of rivers, many aquatic insects that inhabit the headwaters of rivers and therefore are adapted to cold, would undergo a habitat loss [28,66], being gradually replaced by species of middle and lower reaches and generalist species without marked ecological preferences or not linked to a particular habitat [28,40]. Our results confirm what was mentioned above, that projected climate changes with higher temperatures worldwide could lead to cold-water species being excluded by warm-water ones and in the worst cases going extinct [67]. Many freshwater species tend to shift their ranges by moving to higher latitudes and elevations in response to global warming and other related factors [2].

In conclusion, our results show that most of the families studied would shift their distributional range to higher elevations. However, some of them would increase their area of presence when shifting downwards Families linked to lowland areas and with high adaptation capacities and with more generalized habits would be able to move upstream to colonize new sites. However, families living close to mountaintops face the additional risk of going extinct due to their inability to migrate to higher regions [9]. Thus, headwater taxa are expected to undergo a reduction in their distribution area due to global change while being progressively replaced by species from the middle and lower reaches, and by temperature-generalist species [28,40,66], and thus may even disappear.

## Concluding Remarks

The application of predictive MEDPACS approach in three possible scenarios of climate change has shown that an increase of the water temperature, and expected subsequent flow decrease, would have severe repercussions for aquatic macroinvertebrate composition and distribution in the Mediterranean area of Spain and the Balearic Islands. Most of the taxa studied will reduce their distribution area but enlarge their altitudinal range seeking climatically suitable habitats. In conclusion, the composition of macroinvertebrate taxa for Mediterranean rivers in the Iberian Peninsula will vary drastically within these possible new climatic scenarios, at the top of the Mediterranean Mountains and in the southern river basins.

## Supporting Information

S1 File Part 1: Microsoft Access compressed file of the database used for the analyses (it has been divided into three parts because its size). (RAR)

S2 File Part 2: Microsoft Access compressed file of the database used for the analyses (it has been divided into three parts because its size). (RAR)

S3 File Part 3: Microsoft Access compressed file of the database used for the analyses (it has been divided into three parts because its size). (RAR)
